# Association Between VDR and CYP24A1 Polymorphisms, Atopic Dermatitis, and Biochemical Lipid and Vitamin D Profiles in Spanish Population: Case-Control Study

**DOI:** 10.2196/39567

**Published:** 2023-06-27

**Authors:** Ricardo González-Tarancón, Nuria Goñi-Ros, Elvira Salvador-Rupérez, Ángela Hernández-Martín, Silvia Izquierdo-Álvarez, José Puzo-Foncillas, Yolanda Gilaberte-Calzada

**Affiliations:** 1 Department of Clinical Biochemistry Hospital Universitario Miguel Servet Zaragoza Spain; 2 Department of Dermatology Hospital Universitario Niño Jesús Madrid Spain; 3 Department of Clinical Biochemistry Hospital San Jorge Huesca Spain; 4 Department of Dermatology Hospital Universitario Miguel Servet Zaragoza Spain

**Keywords:** VDR, CYP24A1, atopic dermatitis, dermatology, vitamin D, lipid, dermatitis, biochemical, biochemistry, genomics, polymorphism, SNP, Spanish, inflammatory, skin disorder, epidermal, immune, deficiency, children, youth, calcium, phosphorus, risk factor, genetic, development, genotyping, genotype

## Abstract

**Background:**

Atopic dermatitis (AD) is the most prevalent inflammatory skin disorder, characterized by impaired epidermal barrier function and an altered immune response, both of which are influenced by vitamin D deficiency. Single-nucleotide polymorphisms (SNPs) in *VDR* and *CYP24A1* have been previously associated with AD.

**Objective:**

We sought to characterize the associations between the *VDR* and *CYP24A1* polymorphisms and the vitamin D and lipid biochemical profile in children diagnosed with AD.

**Methods:**

A total of 246 participants (143 patients with AD and 103 healthy controls) were enrolled in this study. Genotyping for polymorphisms in *VDR* (rs2239185, rs1544410, rs7975232, rs2238136, rs3782905, rs2239179, rs1540339, rs2107301, rs2239182, and rs731236) and *CYP24A1* (rs2248359 and rs2296241) was performed by allele-specific polymerase chain reaction using integrated fluidic circuit technology. Serum levels of calcium, phosphorus, and vitamin D were measured, and the biochemical lipid profile was determined.

**Results:**

Among *VDR* SNPs, rs2239182 exerted a protective effect against the development of AD, whereas rs2238136 was identified as a risk factor for AD. The GCC haplotype (rs2239185-G, rs1540339-C, and rs2238136-C) appeared to protect against the development of AD. rs2239182-CC was associated with higher 25(OH)D concentrations, whereas rs2238136-TT, rs2239185-GA, and rs2248359-TT were present in a large proportion of patients with serum vitamin D deficiency. rs2239185-AA, rs2239182-CC, and rs1540339-CC were associated with higher serum total cholesterol; rs2239182-TT was associated with lower low-density lipoprotein cholesterol; and rs2239182-TC with lower high-density lipoprotein cholesterol. Both *CYP24A1* SNPs (rs2296241-AA and rs2248359-TT) were associated with higher high-density lipoprotein cholesterol levels.

**Conclusions:**

The *VDR* SNP rs2238136 is a risk factor for AD and other SNPs in *VDR* and *CYP24A1*, which may lead to alterations in biochemical parameters that influence the risk of AD. Our findings highlight the complex genetic basis to AD and indicate that interrelationships between different genetic factors can lead to alterations in vitamin D metabolism or lipid profiles, which in turn may influence the development of AD.

## Introduction

Atopic dermatitis (AD) is the most prevalent inflammatory skin disorder, characterized by impaired epidermal barrier function and an altered immune response; it is caused by a combination of genetic and environmental factors [1-3]. Vitamin D influences both these processes, and several studies have demonstrated an association between 25 OH vitamin D deficiency and AD [4-6], suggesting that vitamin D supplementation may help ameliorate AD severity [7]. However, other authors have highlighted the scarcity of evidence supporting a beneficial effect of vitamin D supplementation in patients with AD [8,9].

The effects of vitamin D deficiency are the consequence of low serum concentrations, mainly due to insufficient sun exposure together with inadequate dietary intake. However, alterations in proteins involved in vitamin D metabolism may also contribute to vitamin D deficiency. Vitamin D receptor (VDR) is an intracellular hormone receptor that specifically binds 1,25(OH)2D3 and mediates its effects. It is encoded by the vitamin D receptor gene (*VDR*; 12q13.11, OMIM#601769), which contains 11 exons and spans approximately 75 kb. Exons 2 and 3 are involved in DNA binding, and exons 7-9 are involved in binding to 1,25(OH)2D3. Using mutation analysis, some authors characterized arg18/arg22, *VDR* residues immediately N-terminal of the first DNA-binding zinc finger, as vital for contact with the general transcription factor II B. Variations in *VDR* (MIM*601769) may alter 1,25(OH)2D3 responsiveness in inflammatory conditions and stimulate the proliferation of T lymphocytes and cytokines [10]. Several studies [11-16] and a recent meta-analysis [17] have described associations between AD and single-nucleotide polymorphisms (SNPs) in *VDR*, although most of these studies only evaluated the impact of the classical FokI (rs2228570), BsmI (rs1544410), ApaI (rs7975232), and TaqI (rs731236) *VDR* polymorphisms.

1,25(OH)2D3 activity also depends on the function of certain enzymes responsible for its endogenous metabolism in keratinocytes and lymphocytes, such as the enzyme 1,25-dihydroxyvitamin D3-24-hydroxylase (encoded by *CYP24A1*, MIM*126065). Vitamin D 24-hydroxylase, encoded by *CYP24A1* (20q13.2, OMIM#126065), is a mitochondrial enzyme responsible for inactivating vitamin D metabolites through the C-24 oxidation pathway. 1,25-(OH)2D3 induces the 24-hydroxylase, whereas hypocalcemia, through increased parathyroid hormone, suppresses this enzyme. The presence of certain SNPs in *CYP24A1* have also been associated with atopic diseases such as AD and asthma [16]. 1,25(OH)2D3 has a short time of action because it induces its own inactivation through the positive regulation of the CYP24A1 enzyme, which carries out the metabolic processes that produce the different products of higher polarity and the loss of hormonal activity. CYP24A1 is located in the inner mitochondrial membrane and is expressed in various tissues at low concentrations, with high capacity to be rapidly activated in response to 1,25(OH)2D3 levels. Particularly, in the liver, this enzyme is not expressed. CYP24A1 activation is not exclusively produced by 1,25(OH)2D3; induction by other compounds such as lithocholic acid, retinoic acids, and pregnane X receptor ligands and by prolonged treatment with different groups of antimicrobial, antituberculosis, and anticonvulsant drugs, among others, has also been demonstrated. CYP24A1 participates in the metabolism of both 25-hydroxy vitamin D3 and 1,25(OH)2D3, although it is more closely linked to the latter [18].

Vitamin D deficiency may also underlie metabolic syndrome, especially during infancy. It has been proposed that in patients with AD, alteration of the lipid profile and the consequent appearance of metabolic syndrome, especially in childhood, may be a consequence of 25 OH vitamin D deficiency due to insufficient serum levels, receptor (VDR) hypofunction, or dysregulation of enzymes involved in its metabolism (CYP24A1) [16,19-21].

In this study, we sought to assess associations between the *VDR* and *CYP24A1* polymorphisms and the 25 OH vitamin D and lipid biochemical profile in children diagnosed with AD.

## Methods

### Study Population

We conducted a case-control pilot study of patients aged between 2 months and 14 years who fulfilled the following criteria: AD at the time of inclusion, skin phototype 2-4, Mediterranean phenotype, and born to parents of Spanish origin. Participants were recruited between January 2011 and December 2012 in the Departments of Dermatology and Paediatric Allergy of the San Jorge Hospital (Huesca, Spain), primary care centers in Huesca city, and the Dermatology Department of the Niño Jesús Hospital (Madrid, Spain). These patients were initially recruited for other studies previously published by our group [22,23]. Participants in the control group were recruited at the University Hospital Miguel Servet (Zaragoza, Spain), being apparently healthy individuals, and by using the following exclusion criteria: family history of atopy or allergic diseases; symptoms of atopic eczema, asthma, or hay fever; and allergies to food, pollen, or other environmental allergens (eg, animals).

AD severity was scored using the Scoring Atopic Dermatitis (SCORAD) index [24,25] and classified as mild (SCORAD <15), moderate (SCORAD 15-40), or severe (SCORAD >40) [26]. Personal history of atopic diseases, including asthma and allergic rhinitis, was also recorded.

### Ethics Approval

This study was approved by the Aragon Ethical Committee for Clinical Research (PI08/81). Written informed consent was obtained from all participants, or their guardians, before inclusion.

### Genomic Studies

Genomic DNA was obtained from peripheral blood leukocytes extracted from ethylenediaminetetraacetic acid whole blood samples using the QIAamp DNA Blood Mini Kit (QIAGEN) and an automated EZ1 biorobot (QIAGEN). Both cohorts were genotyped for the *VDR* (rs2239185, rs1544410, rs7975232, rs2238136, rs3782905, rs2239179, rs1540339, rs2107301, rs2239182, and rs731236) and *CYP24A1* (rs2248359 and rs2296241) polymorphisms.

SNP genotyping was performed using the commercial FlexSix Genotyping integrated fluidic circuit kit (Fluidigm). This method is based on allele-specific polymerase chain reaction detection using an integrated fluidic circuit.

### Biochemical Studies

Calcium (mg/dL), total cholesterol (mg/dL), high-density lipoprotein (HDL) cholesterol (mg/dL), triglycerides (mg/dL), and phosphorus (mg/dL) serum concentrations were measured by spectrophotometric methods, and 25(OH)D (ng/mL), immunoglobulin E (UI/L), and parathyroid hormone (pg/mL) by immunochemiluminescence using an automated AU5400 and UniCel DxI 800 (Beckman Coulter), respectively. While acknowledging that the gold standard for vitamin D metabolites is liquid chromatography-mass spectrometry, immunochemiluminescent assays are the most widely used clinically. Low-density lipoprotein (LDL) cholesterol (mg/dL) were calculated by the Friedewald equation.

### Data Analyses

Descriptive statistics for quantitative values were expressed as the mean and SD, according to the data distribution. Categorical variables were presented as frequencies and percentages. Chi-square or Fisher exact tests were used to assess associations between the *VDR* and *CYP24A1* polymorphisms and AD and AD-associated variables, including the SCORAD index and associated atopic diseases. The association between genotypes or alleles and AD was assessed by calculating the odds ratio (OR) and 95% CIs. Allele and genotype frequencies were calculated by direct counting, and the chi-square test was used to compare frequencies between cases and controls. The Hardy-Weinberg equilibrium was tested in both groups using the chi-square goodness-of-fit test. The level of statistical significance was set at *P*<.05. Statistical analyses were performed using SPSS version 19 (IBM Corp) and R software (haplo.stats package; R Foundation for Statistical Computing).

## Results

The clinical characteristics of the study population is described in Table 1.

Genotype distributions of *VDR* and *CY24A1* SNPs in the control and AD groups are summarized in Multimedia Appendix 1. rs7975232 and rs2107301 were among the SNPs initially proposed for evaluation but were later excluded as they failed to meet Hardy-Weinberg equilibrium criteria. Genotype frequencies for global (TOT, n=2504), European (EUR, n=503), and Iberic populations (IBS, n=107), based on 1000 Genomes Project phase 3 populations, are also presented for the sake of comparison [27]*.*

Table 2 summarizes the association between AD and SNPs. For each SNP, the results for the 5 genetic models (codominant, dominant, recessive, overdominant, and additive) are shown. Statistically significant associations were observed for 2 polymorphisms in *VDR*: rs2239182, which was identified as a protective factor; and rs2238136, which was identified as a risk factor. No significant associations were observed for the remaining polymorphisms analyzed.

The presence of rs2239182-C/C in the codominant model exerted a protective effect, reducing the risk of AD by 66% (OR 0.34, 95% CI 0.13-0.87; *P*=.03), whereas the presence of allele C in the dominant model reduced the risk by 58% (OR 0.42, 95% CI 0.18-0.90; *P*=.03). Moreover, rs2238136 was identified as a risk factor for AD in the codominant, dominant, and overdominant models (*P*=.02, .02, and .02, respectively). These data indicate that in the overdominant and codominant models, the risk of AD increases 2.94- and 3.09-fold, respectively, in patients with the rs2238136-C/T genotype. In the dominant model, the presence of one T allele increased the risk of AD 2.7-fold (OR 2.70, 95% CI 1.24-6.15; *P*=.02).

The association between the *VDR* and *CYP24A1* genotypes and the severity of AD and other atopic diseases is shown in Table 3. We observed no significant association between any of the SNPs analyzed and the severity of AD (SCORAD index) or other atopic diseases, such as asthma or rhinitis.

The association between AD and specific combinations of alleles and haplotypes in selected SNPs is shown in Table 4. rs2238136, rs2239185, and rs1540339 were selected owing to their presence in intronic regions of *VDR* that encode transcription factors. rs2239182, rs2107301, and 2239179 are located in the same intronic region (between exons 4 and 5) and were therefore selected given the possibility that these SNPs are in linkage equilibrium. Finally, haplotypes corresponding to combinations of SNPs in *CYP24A1* were also analyzed (Table 4). Significant associations were observed only for the GCC haplotype (rs2239185-G, rs1540339-C, and rs2238136-C), which appeared to exert a protective effect, reducing the risk of AD by 49% (OR 0.511, 95% CI 0.232-0.939; *P*=.04).

The associations between SNP genotypes and serum levels of calcium phosphorus and vitamin D (Table 5) and lipid profile (Table 6) were also analyzed, considering cases and controls as a single cohort. The data showed that 25(OH)D concentrations were higher in individuals with the rs2239182-CC versus the rs2239182-TT genotype (28.10 ± 8.80 vs 24.80 ± 8.70 ng/mL; *P*=.03), whereas vitamin D levels were lower in individuals with the rs2238136-TT genotype than the rs2238136-CT or rs2238136-CC genotype (21.82, 27.06, and 29.04 ng/mL, respectively; *P*=.046; Table 5).

rs2239185-GA and rs2248359-TT were present in a large proportion of patients with serum 25 OH vitamin D deficiency (42.31% and 56.52%, respectively) compared with alternative genotypes. The presence of a T allele in rs2239179 was associated with lower levels of phosphorus compared with the CC haplotype (5.11 vs 6.75 mg/dL; *P*=.04) (Table 5).

rs2239185-AA, rs2239182-CC, and rs1540339-CC were associated with higher total cholesterol concentrations; rs2239182-TT was associated with lower LDL cholesterol; and rs2239182-TC with lower HDL cholesterol. Both *CYP24A1* SNPs (rs2296241-AA and rs2248359-TT) were associated with higher HDL cholesterol levels (Table 6).

**Table 1:** Clinical characteristics of the study population (N=246).

Characteristics		AD^a^ cases		Controls
Group size, n		143		103
Age (years), mean (SD)^b^		5.43 (3.98)		8.93 (5.81)
Sex (male/female)^c^, n		73/70		57/46
**Severity (SCORAD^d^ index), n (%)**				
	Mild^e^	69 (48.3)	—^f^	
	Moderate^g^	61 (42.7)	—	
	Severe^h^	13 (9)	—	
Asthma, n (%)		44 (34.6)		—
Rhinitis, n (%)		28 (22)		—
Asthma+rhinitis, n (%)		21 (16.5)		—

^a^AD: atopic dermatitis.

^b^*P*<.05.

^c^*P*=.50.

^d^SCORAD: Scoring Atopic Dermatitis.

^e^Mild: <15.

^f^Not available.

^g^Moderate: 15-40.

^h^Severe: >40.

**Table 2:** Association between the VDR and CYP24A1 polymorphisms and atopic dermatitis.

SNP^a^/model			Overall, n (%)	Control, n (%)	Atopic dermatitis, n (%)	OR^b^ (95% CI)	*P* value^c^	*P* value^d^
**rs731236**								
	**Codominant**		191	77	114			N/A^e^
		A/A	81 (42.4)	37 (48)	44 (38.6)	N/A	N/A	.35
		G/A	75 (39.3)	25 (33)	50 (43.9)	1.64 (0.82-3.34)	.17	N/A
		G/G	35 (18.3)	15 (20)	20 (17.5)	1.09 (0.47-2.64)	.84	N/A
	**Dominant**		191	77	114			N/A
		A/A	81 (42.4)	37 (48)	44 (38.6)	N/A	N/A	.33
		G/A-G/G	110 (57.6)	40 (52)	70 (61.4)	1.44 (0.76-2.71)	.26	N/A
	**Recessive**		191	77	114			N/A
		A/A-G/A	156 (81.7)	62 (81)	94 (82.5)	N/A	N/A	.89
		G/G	35 (18.3)	15(19)	20 (17.5)	0.87 (0.39-1.98)	.73	N/A
	**Overdominant**		191	77	114			N/A
		A/A-G/G	116 (60.7)	52 (68)	64 (56.1)	N/A	N/A	.21
		G/A	75 (39.3)	25 (32)	50 (43.9)	1.59 (0.84-3.10)	.16	N/A
	**Additive**		0.77 (0.74)	0.72 (0.78)	0.79 (0.72)	1.13 (0.74-1.73)	.56	.57
**rs1544410**								
	**Codominant**		188	71	117			N/A
		C/C	67 (35.6)	27 (38)	40 (34.2)	N/A	N/A	.77
		C/T	83 (44.2)	29 (41)	54 (46.2)	1.25 (0.64-2.45)	.51	N/A
		T/T	38 (20.2)	15 (21)	23 (19.7)	1.03 (0.46-2.37)	.94	N/A
	**Dominant**		188	71	117			N/A
		C/C	67 (35.6)	27 (38)	40 (34.2)	N/A	N/A	.71
		C/T-T/T	121 (64.4)	44 (62)	77 (65.8)	1.18 (0.64-2.18)	.60	N/A
	**Recessive**		188	71	117			N/A
		C/C-C/T	150 (79.8)	56 (79)	94 (80.3)	N/A	N/A	.96
		T/T	38 (20.2)	15 (21)	23 (19.7)	0.91 (0.44-1.93)	.81	N/A
	**Overdominant**		188	71	117			N/A
		C/C-T/T	105 (55.9)	42 (59)	63 (53.9)	N/A	N/A	.58
		C/T	83 (44.2)	29 (41)	54 (46.2)	1.24 (0.68-2.27)	.48	N/A
	**Additive**		0.85 (0.73)	0.83 (0.76)	0.85 (0.72)	1.05 (0.70-1.57)	.83	.83
**rs2239185**								
	**Codominant**		202	89	113			N/A
		A/A	65 (32.2)	29 (32)	36 (31.9)	N/A	N/A	.52
		G/A	77 (38.1)	30 (34)	47 (41.6)	1.24 (0.57-2.68)	.59	N/A
		G/G	60 (29.7)	30 (34)	30 (26.6)	0.79 (0.36-1.77)	.57	N/A
	**Dominant**		202	89	113			N/A
		A/A	65 (32.2)	29 (33)	36 (31.9)	N/A	N/A	>.99
		G/A-G/G	137 (67.8)	60 (67)	77 (68.1)	1.02 (0.51-1.99)	.96	N/A
	**Recessive**		202	89	113			N/A
		A/A-G/A	142 (70.3)	59 (66)	83 (73.5)	N/A	N/A	.41
		G/G	60 (29.7)	30 (34)	30 (26.6)	0.71 (0.36-1.41)	.32	N/A
	**Overdominant**		202	89	113			N/A
		A/A-G/G	125 (61.9)	59 (66)	66 (58.4)	N/A	N/A	.41
		G/A	77 (38.1)	30 (34)	47 (41.6)	1.38 (0.72-2.71)	.33	N/A
	**Additive**		0.97 (0.78)	1.02 (0.82)	0.95 (0.77)	0.89 (0.60-1.33)	.58	.59
**rs2239182**								
	**Codominant**		205	85	120			N/A
		T/T	69 (33.7)	24 (28)	45 (37.5)	N/A	N/A	.04^e^
		T/C	90 (43.9)	38 (45)	52 (43.3)	0.47 (0.20-1.08)	.08	N/A
		C/C	46 (22.4)	23 (27)	23 (19.2)	0.34 (0.13-0.87)	.03^e^	N/A
	**Dominant**		205	85	120			N/A
		T/T	69 (33.7)	24 (28)	49 (40.8)	N/A	N/A	.046^e^
		T/C-C/C	136 (66.3)	61 (72)	71 (59.2)	0.42 (0.18-0.90)	.03^e^	N/A
	**Recessive**		205	85	120			N/A
		T/T-T/C	159 (77.6)	62 (73)	97 (80.8)	N/A	N/A	.24
		C/C	46 (22.4)	23 (27)	23 (19.2)	0.64 (0.33-1.25)	.19	N/A
	**Overdominant**		205	85	120			N/A
		T/T-C/C	115 (56.1)	47 (55)	68 (56.7)	N/A	N/A	.96
		T/C	90 (43.9)	38 (45)	52 (43.3)	0.95 (0.54-1.66)	.85	N/A
	**Additive**		0.89 (0.74)	0.99 (0.75)	0.82 (0.73)	0.73 (0.50-1.07)	.10	.11
**rs1540339**								
	**Codominant**		198	82	116			N/A
		C/C	76 (38.4)	32 (40)	44 (37.9)	N/A	N/A	.82
		T/C	82 (41.4)	35 (43)	47 (40.5)	0.99 (0.51-1.95)	.99	N/A
		T/T	40 (20.2)	15 (18)	25 (21.6)	1.27 (0.55-3.03)	.58	N/A
	**Dominant**		198	82	116			N/A
		C/C	76 (38.4)	32 (39)	44 (37.9)	N/A	N/A	.94
		T/C-T/T	122 (61.6)	50 (61)	72 (62.1)	1.08 (0.58-1.99)	.81	N/A
	**Recessive**		198	82	116			N/A
		C/C-T/C	158 (79.8)	67 (82)	91 (78.5)	N/A	N/A	.66
		T/T	40 (20.2)	15 (18)	25 (21.6)	1.27 (0.60-2.83)	.54	N/A
	**Overdominant**		198	82	116			N/A
		C/C-T/T	116 (58.6)	47 (57)	69 (59.5)	N/A	N/A	.90
		T/C	82 (41.4)	35 (43)	47 (40.5)	0.92 (0.50-1.69)	.78	N/A
	**Additive**		0.82 (0.75)	0.78 (0.73)	0.84 (0.76)	1.11 (0.74-1.66)	.62	.62
**rs2239179**								
	**Codominant**		193	80	113			N/A
		T/T	84 (43.5)	36 (45)	48 (42.5)	N/A	N/A	.43
		T/C	86 (44.6)	32 (40)	54 (47.8)	1.27 (0.64-2.53)	.50	N/A
		C/C	23 (11.9)	12 (15)	11 (9.7)	0.66 (0.24-1.87)	.43	N/A
	**Dominant**		193	80	113			N/A
		T/T	84 (43.5)	36 (45)	48 (42.5)	N/A	N/A	.90
		T/C-C/C	109 (56.5)	44 (55)	65 (57.5)	1.10 (0.58-2.09)	.77	N/A
	**Recessive**		193	80	113			N/A
		T/T-T/C	170 (88.1)	68 (85)	102 (90.3)	N/A	N/A	.39
		C/C	23 (11.9)	12 (15)	11 (9.7)	0.59 (0.23-1.57)	.28	N/A
	**Overdominant**		193	80	113			N/A
		T/T-C/C	107 (55.4)	48 (60)	59 (52.2)	N/A	N/A	.40
		T/C	86 (44.6)	32 (40)	54 (47.8)	1.39 (0.73-2.67)	.32	N/A
	**Additive**		0.68 (0.67)	0.71 (0.73)	0.67 (0.65)	0.93 (0.58-1.48)	.75	.76
**rs3782905**								
	**Codominant**		206	87	119			.62
		C/C	113 (54.9)	47 (54)	66 (55.5)	N/A	N/A	N/A
		C/G	74 (35.9)	30 (34)	44 (37)	1.04 (0.57-1.91)	.89	N/A
		G/G	19 (9.2)	10 (11)	9 (7.6)	0.64 (0.24-1.74)	.38	N/A
	**Dominant**		206	87	119			.95
		C/C	113 (54.9)	47 (54)	66 (55.5)	N/A	N/A	N/A
		C/G-G/G	93 (45.2)	40 (46)	53 (44.5)	0.94 (0.54-1.65)	.84	N/A
	**Recessive**		206	87	119			.47
		C/C-C/G	187 (90.8)	77 (89)	110 (92.4)	N/A	N/A	N/A
		G/G	19 (9.2)	10 (11)	9 (7.6)	0.63 (0.24-1.66)	.35	N/A
	**Overdominant**		206	87	119			.83
		C/C-G/G	132 (64.1)	57 (66)	75 (63)	N/A	N/A	N/A
		C/G	74 (35.9)	30 (34)	44 (37)	1.11 (0.62-2.00)	.72	N/A
	**Additive**		0.54 (0.66)	0.57 (0.69)	0.52 (0.64)	0.88 (0.58-1.34)	.56	.57
**rs2238136**								
	**Codominant**		178	74	104			N/A
		C/C	95 (53.4)	50 (68)	45 (43.3)	N/A	N/A	.02^e^
		C/T	69 (38.8)	18 (24)	51 (49)	3.09 (1.34-7.73)	.008^e^	N/A
		T/T	14 (7.9)	6 (8)	8 (7.7)	1.44 (0.37-7.41)	.62	N/A
	**Dominant**		178	74	104			N/A
		C/C	95 (53.4)	50 (68)	45 (43.3)	N/A	N/A	.02^e^
		C/T-T/T	83 (46.6)	24 (32)	59 (56.7)	2.70 (1.24-6.15)	.01^e^	N/A
	**Recessive**		178	74	104			N/A
		C/C-C/T	164 (92.1)	68 (92)	96 (92.3)	N/A	N/A	>.99
		T/T	14 (7.9)	6 (8)	8 (7.7)	0.92 (0.24-4.62)	.91	N/A
	**Overdominant**		178	74	104			N/A
		C/C-T/T	109 (61.2)	56 (76)	53 (51)	N/A	N/A	.02^e^
		C/T	69 (38.8)	18 (24)	51 (49)	2.94 (1.30-7.24)	.009^e^	N/A
	**Additive**		0.58 (0.63)	0.41 (0.64)	0.64 (0.62)	1.92 (1.00-3.71)	.05	.06
**rs2296241**								
	**Codominant**		192	100	92			N/A
		A/A	66 (34.4)	39 (39)	27 (29)	N/A	N/A	.63
		G/A	87 (45.3)	44 (44)	43 (47)	1.39 (0.50-3.88)	.53	N/A
		G/G	39 (20.3)	17 (17)	22 (24)	1.75 (0.51-6.33)	.38	N/A
	**Dominant**		192	100	92			N/A
		A/A	66 (34.4)	39 (39)	27 (29)	N/A	N/A	.53
		G/A-G/G	126 (65.6)	61 (61)	65 (71)	1.50 (0.59-3.90)	.40	N/A
	**Recessive**		192	100	92			N/A
		A/A-G/A	153 (79.7)	83 (83)	70 (76)	N/A	N/A	.67
		G/G	39 (20.3)	17 (17)	22 (24)	1.46 (0.49-4.57)	.50	N/A
	**Overdominant**		192	100	92			N/A
		A/A-G/G	105 (54.7)	56 (56)	49 (53)	N/A	N/A	.98
		G/A	87 (45.3)	44 (44)	43 (47)	1.12 (0.46-2.73)	.81	N/A
	**Additive**		0.88 (0.74)	0.79 (0.73)	0.95 (0.74)	1.34 (0.73-2.46)	.34	.35
**rs2248359**								
	**Codominant**		193	96	97			.89
		C/C	68 (35.2)	33 (34)	35 (36)	N/A	N/A	N/A
		C/T	76 (39.4)	36 (38)	40 (41)	1.05 (0.34-3.17)	.92	N/A
		T/T	49 (25.4)	27 (28)	22 (23)	0.79 (0.23-2.73)	.71	N/A
	**Dominant**		193	96	97			>.99
		C/C	68 (35.2)	33 (34)	35 (36)	N/A	N/A	N/A
		C/T-T/T	125 (64.8)	63 (66)	62 (64)	0.94 (0.34-2.53)	.90	N/A
	**Recessive**		193	96	97			.83
		C/C-C/T	144 (74.6)	69 (72)	75 (77)	N/A	N/A	N/A
		T/T	49 (25.4)	27 (28)	22 (23)	0.77 (0.26-2.31)	.64	N/A
	**Overdominant**		193	96	97			.95
		C/C-T/T	117 (60.6)	60 (63)	57 (59)	N/A	N/A	N/A
		C/T	76 (39.4)	36 (38)	40 (41)	1.16 (0.44-3.08)	.77	N/A
	**Additive**		0.90 (0.78)	0.94 (0.80)	0.87 (0.77)	0.90 (0.49-1.64)	.72	.73

^a^SNP: single-nucleotide polymorphism.

^b^OR: odds ratio.

^c^*P* value (comparison with the null model).

^d^*P* value for the null model.

^e^Statistically significant (*P*<.05).

**Table 3:** Association between the VDR and CYP24A1 polymorphisms and the severity of atopic dermatitis, asthma, rhinitis, and asthma+rhinitis.^a^

SNP^b^	Atopic dermatitis severity	Asthma	Rhinitis	Asthma+rhinitis
rs731236	.84	.36	.23	.12
rs1544410	.56	.79	.21	.10
rs2239185	.32	.21	.17	.12
rs2239182	.44	.30	.18	.15
rs1540339	.86	.43	.12	.14
rs2239179	.42	.80	.21	.35
rs3782905	.10	.29	.71	.22
rs2238136	.82	.52	.25	.55
rs2296241	.54	.66	.90	.78
rs2248359	.97	.69	.21	.32

^a^*P* value is shown for each group. Atopic dermatitis severity is based on Scoring Atopic Dermatitis (SCORAD). This table only corresponds to the codominant model for each SNP, regardless of whether this model is the best fit for the distribution.

^b^SNP: single-nucleotide polymorphism.

**Table 4:** Haplotypes for combination of selected polymorphisms.

SNP^a^ 1	SNP 2	SNP 3	F^b^	OR^c^ (95% CI)	*P* value
rs2239185	rs1540339	rs2238136	N/A^d^	N/A	N/A
A	C	C	0.262	1	N/A
A	C	T	0.090	0.830 (0.270-2.546)	.74
A	T	C	0.091	0.474 (0.175-1.279)	.14
A	T	T	0.072	2.882 (0.634-13.097)	.17
G	C	C	0.208	0.511 (0.232-0.939)	.04^e^
G	C	T	0.032	0.686 (0.240-2.409)	.57
G	T	C	0.166	0.933 (0.441-1.974)	.86
G	T	T	0.079	1.252 (0.411-3.812)	.69
rs2239182	rs2107301	rs2239179	N/A	N/A	N/A
C	G	C	0.347	1	N/A
C	A	C	0.010	80.403 (80.285-80.521)	<.001
C	G	T	0.087	0.752 (0.323-1.754)	.51
T	A	T	0.284	1.555 (0.726-3.334)	.26
T	G	T	0.267	1.053 (0.539-2.059)	.88
rs2248359	rs2296241	N/A	N/A	N/A	N/A
T	A	N/A	0.398	1	N/A
C	A	N/A	0.138	1.620 (0.552-4.751)	.38
C	G	N/A	0.398	1.108 (0.546-2.247)	.78
T	G	N/A	0.065	0.904 (0.189-4.323)	.90

^a^SNP: single-nucleotide polymorphism.

^b^F: haplotype frequency.

^c^OR: odds ratio.

^d^N/A: not applicable.

^e^Statistically significant (*P*<.05).

**Table 5:** Association between the VDR and CYP24A1 polymorphisms and selected calcium phosphorus metabolism and vitamin D–related analytes.

SNP^a^/genotype			25(OH)D (ng/mL), mean (SD)		*P* value		25(OH)D >30 ng/mL (%)		25(OH)D 20-29 ng/mL (%)		25(OH)D <20 ng/mL (%)		*P* value		Calcium (mg/dL), mean (SD)		*P* value		Phosphorus (mg/dL), mean (SD)		*P* value		PTH^b^ (pg/mL), mean (SD)		*P* value
**rs731236**																									
	G/G	28.35 (12.28)		N/A^c^		28.57		33.33		38.10		.44		9.79 (0.59)		N/A		5.34 (1.24)		N/A		28.67 (16.17)		N/A	
	G/A	27.81 (11.20)		.86		24.07		35.19		40.74		N/A		9.84 (0.50)		.69		5.01 (0.65)		.69		24.38 (14.34)		.30	
	A/A	28.05 (12.82)		.92		18.18		52.27		29.55		N/A		9.78 (0.57)		.99		5.97 (5.10)		.39		29.55 (17.15)		.82	
**rs1544410**																									
	T/T	29.51 (11.10)		N/A		16.67		41.67		41.67		.23		9.83 (0.55)		N/A		4.98 (0.84)		N/A		29.06 (16.98)		N/A	
	C/C	29.35 (13.74)		.96		14.63		51.22		34.15		N/A		9.89 (0.55)		.65		5.42 (1.18)		.59		28.06 (15.62)		.82	
	C/T	26.42 (10.55)		.28		31.03		32.76		36.21		N/A		9.71 (0.54)		.33		5.68 (4.80)		.37		27.43 (16.37)		.69	
**rs2239185**																									
	G/G	29.60 (14.91)		N/A		20.00		46.67		33.33		.049^d^		9.75 (0.58)		N/A		4.89 (0.86)		N/A		27.14 (16.42)		N/A	
	A/A	27.45 (9.15)		.47		13.89		55.56		30.56		N/A		9.78 (0.55)		.83		5.85 (5.03)		.26		26.94 (14.88)		.96	
	G/A	27.49 (11.93)		.44		30.77		26.92		42.31		N/A		9.84 (0.56)		.53		5.35 (1.11)		.60		29.52 (17.75)		.59	
**rs2239182**																									
	C/C	28.10 (8.80)		N/A		20.83		37.50		41.67		.48		9.84 (0.49)		N/A		5.18 (0.47)		N/A		28.57 (14.76)		N/A	
	T/T	24.80 (8.70)		.03^d^		25.00		47.73		27.27		N/A		9.69 (0.57)		.37		4.93 (0.71)		.82		29.03 (17.86)		.94	
	T/C	30.00 (13.50)		.26		20.69		34.48		44.83		N/A		9.85 (0.52)		.98		5.77 (4.35)		.58		27.33 (15.20)		.83	
**rs1540339**																									
	T/T	25.61 (10.01)		N/A		24.00		44.00		32.00		.80		9.90 (0.50)		N/A		5.06 (0.74)		N/A		25.53 (12.72)		N/A	
	C/C	28.90 (13.65)		.27		25.53		34.04		40.43		N/A		9.87 (0.54)		.86		6.11 (5.19)		.31		27.42 (14.51)		.73	
	T/C	28.27 (10.91)		.36		20.00		46.00		34.00		N/A		9.70 (0.56)		.21		5.09 (0.72)		.98		28.49 (18.11)		.58	
**rs2239179**																									
	C/C	27.35 (7.60)		N/A		18.18		45.45		36.36		.45		9.89 (0.64)		N/A		6.75 (7.12)		N/A		28.30 (15.29)		N/A	
	T/T	26.99 (9.89)		.93		18.75		50.00		31.25		N/A		9.73 (0.54)		.22		5.11 (0.75)		.04^d^		26.86 (17.90)		.73	
	T/C	28.97 (14.05)		.68		27.12		32.20		40.68		N/A		9.80 (0.51)		.46		5.11 (0.75)		.03^d^		28.92 (15.17)		.88	
**rs3782905**																									
	C/C	29.90 (9.88)		N/A		22.22		33.33		44.44		.21		9.86 (0.45)		N/A		5.00 (0.67)		N/A		26.03 (13.97)		N/A	
	C/G	26.92 (12.59)		.51		32.61		32.61		34.78		N/A		9.82 (0.57)		.72		6.10 (5.51)		.15		27.50 (15.48)		.73	
	C/C	29.70 (12.21)		.96		14.29		45.71		40.00		N/A		9.74 (0.56)		.30		5.11 (0.82)		.88		29.57 (17.44)		.38	
**rs2238136**																									
	T/T	21.82 (11.46)		N/A		50.00		37.50		12.50		.14		9.81 (0.66)		N/A		5.79 (1.74)		N/A		28.96 (15.27)		N/A	
	C/T	27.06 (9.85)		.18		20.75		47.17		32.08		N/A		9.90 (0.55)		.65		5.22 (0.91)		.10		29.06 (14.94)		.99	
	C/C	29.04 (10.26)		.046^d^		19.15		34.04		46.81		N/A		9.73 (0.53)		.69		5.25 (0.71)		.12		24.21 (13.41)		.39	
**rs2296241**																									
	G/G	28.85 (13.97)		N/A		18.18		54.55		27.27		.10		9.33 (0.84)		N/A		8.94 (12.02)		N/A		38.05 (10.43)		N/A	
	A/A	31.54 (8.26)		.58		14.29		14.29		71.43		N/A		9.59 (0.44)		.22		4.86 (0.48)		.05		32.42 (15.21)		.36	
	G/A	26.62 (12.75)		.62		25.00		45.00		30.00		N/A		9.71 (0.51)		.07		5.03 (0.76)		.06		30.71 (17.67)		.24	
**rs2248359**																									
	T/T	27.82 (11.60)		N/A		43.48		0.00		56.52		.007^d^		9.72 (0.48)		N/A		5.02 (0.78)		N/A		36.43 (16.64)		N/A	
	C/C	29.24 (17.95)		.81		18.07		48.19		33.73		N/A		9.30 (0.80)		.06		7.82 (10.17)		.18		33.88 (10.92)		.71	
	C/T	29.30 (10.96)		.80		11.76		52.94		35.29		N/A		9.63 (0.44)		.67		4.78 (0.66)		.90		30.27 (17.24)		.31	

^a^SNP: single-nucleotide polymorphism.

^b^PTH: parathyroid hormone.

^c^N/A: not applicable.

^d^Statistically significant (*P*<.05).

**Table 6:** Association between the VDR and CYP24A1 polymorphisms and selected lipid analytes.

SNP^a^/genotype		Cholesterol (mg/dL), mean (SD)	*P* value	LDL^b^-cholesterol (mg/dL), mean (SD)	*P* value	HDL^c^-cholesterol (mg/dL), mean (SD)	*P* value	Triglycerides (mg/dL), mean (SD)	*P* value
**rs731236**									
	G/G	171.22 (29.50)	N/A^d^	108.60 (28.86)	N/A	52.42 (12.14)	N/A	83.22 (41.04)	N/A
	G/A	168.54 (31.48)	.69	106.74 (29.41)	.81	52.77 (12.89)	.91	79.59 (55.10)	.76
	A/A	162.17 (25.26)	.18	99.57 (27.37)	.26	52.54 (9.73)	.97	74.21 (50.49)	.45
**rs1544410**									
	T/T	170.26 (24.58)	N/A	104.50 (24.24)	N/A	53.70 (12.76)	N/A	79.71 (40.48)	N/A
	C/C	163.48 (25.51)	.29	100.02 (27.26)	.54	53.70 (10.50)	>.99	74.52 (48.66)	.64
	C/T	166.60 (32.16)	.56	107.30 (29.96)	.70	51.71 (11.61)	.50	78.55 (54.30)	.92
**rs2239185**									
	G/G	160.71 (23.63)	N/A	98.69 (22.29)	N/A	53.75 (9.55)	N/A	78.08 (54.82)	N/A
	A/A	176.78 (33.68)	.04^e^	112.98 (33.50)	.06	53.69 (12.16)	.98	74.44 (37.65)	.75
	G/A	166.04 (27.54)	.37	101.06 (26.51)	.74	51.41 (12.23)	.43	82.05 (57.35)	.71
**rs2239182**									
	C/C	175.97 (26.76)	N/A	113.83 (28.55)	N/A	57.68 (11.79)	N/A	69.92 (29.82)	N/A
	T/T	156.37 (26.51)	.009^e^	98.22 (25.90)	.02^e^	52.73 (12.45)	.08	80.45 (55.17)	.31
	T/C	166.30 (28.78)	.09	104.27 (26.77)	.15	51.63 (9.59)	.03^e^	77.18 (48.93)	.46
**rs1540339**									
	T/T	158.26 (22.55)	N/A	96.33 (20.72)	N/A	52.09 (13.04)	N/A	89.63 (59.93)	N/A
	C/C	171.58 (30.87)	.04^e^	108.44 (31.12)	.10	53.12 (11.77)	.73	73.63 (46.83)	.16
	T/C	164.70 (28.06)	.30	103.48 (27.68)	.33	53.02 (10.43)	.76	74.66 (46.86)	.18
**rs2239179**									
	C/C	177.07 (23.27)	N/A	109.16 (25.11)	N/A	57.18 (15.64)	N/A	82.67 (37.52)	N/A
	T/T	162.59 (27.57)	.08	100.68 (26.66)	.37	51.48 (11.98)	.14	80.10 (52.39)	.86
	T/C	168.03 (30.59)	.27	107.50 (30.38)	.86	52.63 (9.67)	.24	76.58 (52.48)	.68
**rs3782905**									
	G/G	168.27 (23.06)	N/A	104.48 (21.22)	N/A	53.67 (14.65)	N/A	75.33 (32.11)	N/A
	C/G	169.19 (26.91)	.91	104.96 (22.69)	.96	54.82 (10.33)	.76	73.74 (42.84)	.90
	C/C	164.15 (29.37)	.60	103.07 (30.00)	.87	52.58 (11.15)	.76	76.35 (46.56)	.93
**rs2238136**									
	T/T	168.33 (40.99)	N/A	109.70 (46.48)	N/A	52.00 (18.19)	N/A	100.22 (44.92)	N/A
	C/T	165.28 (26.98)	.78	104.77 (25.96)	.70	51.74 (11.82)	.96	76.46 (55.76)	.22
	C/C	166.97 (31.15)	.90	104.33 (30.19)	.68	52.65 (11.19)	.90	79.71 (51.44)	.28
**rs2296241**									
	G/G	160.08 (27.36)	N/A	104.75 (31.55)	N/A	48.00 (11.75)	N/A	88.50 (46.87)	N/A
	A/A	163.56 (27.81)	.70	102.58 (19.48)	.86	61.50 (8.83)	.048^e^	88.00 (49.27)	.98
	G/A	162.31 (18.36)	.79	95.11 (12.89)	.46	52.57 (12.59)	.51	103.96 (64.99)	.45
**rs2248359**									
	T/T	163.82 (28.39)	N/A	110.87 (24.42)	N/A	70.33 (6.66)	N/A	101.45 (55.99)	N/A
	C/C	159.50 (23.03)	.63	98.12 (21.07)	.39	52.25 (9.74)	.02^e^	82.75 (46.58)	.38
	C/T	162.06 (20.49)	.85	94.52 (19.82)	.31	46.40 (10.71)	.005^e^	114.82 (65.83)	.54

^a^SNP: single nucleotide polymorphism.

^b^LDL: low-density lipoprotein.

^c^HDL: high-density lipoprotein.

^d^N/A: not applicable.

^e^Statistically significant (*P*<.05).

## Discussion

Previous studies have reported associations between the development of AD and certain SNPs such as *FokI* (rs2228570), *BsmI* (rs1544410), *ApaI* (rs7975232), and *TaqI* (rs731236) [11-15]. Contradicting some of those findings, a recent meta-analysis found that only rs1544410 and rs731236 were associated with the development of AD and asthma [17]. In this study, we found an association between AD and a total of 12 selected SNPs (10 in *VDR* and 2 in *CYP24A1*). Although a previous study analyzed 13 SNPs in *VDR*, only the association with asthma was investigated [28]. Therefore, ours is one of the most extensive studies performed to date in terms of the number of SNPs analyzed in these 2 genes implicated in AD.

Although our findings do not support the association between AD and classic SNPs described in the literature (rs1544410 and rs731236), we observed a significant association between AD and the polymorphisms rs2238136 and rs2239182. Both SNPs correspond to intronic regions of *VDR*, and none have been previously described as risk factors for AD.

Given their impact on keratinocyte proliferation and differentiation in the epidermal layer, SNPs in genes involved in vitamin D metabolism may be important genetic risk factors for the development of AD [29]. Our data suggest that altered vitamin D metabolism due to the presence of genetic variants influences the pathogenesis of AD. In this way, we identified rs2239182 and the GCC haplotype (rs2239185-G, rs1540339-C, and rs2238136-C) as protective factors and rs2238136 as a risk factor.

rs2238136 is located in an intronic region between exons 1 and 2 of *VDR*. Although no clinical information regarding the functionality of the encoded protein is reported in the consulted databases (ClinVar, Ensembl, PubMed, and ClinGen), the location of this SNP in a transcription factor binding region (GATA1) suggests that it could regulate gene expression and alter protein function. SNP databases (ClinVar and Ensmbl) contain no information about the association between this SNP and AD. Therefore, this is the first study to provide evidence supporting an association between rs2238136 and AD.

rs2239182 is also located in an intronic region (between exons 5 and 6 of *VDR)*. It is mentioned in 18 studies cited in ClinVar and 22 in Ensmbl, but none report any association with AD. Because ours is the first study investigating this association, we cannot compare our findings with those of previously published studies.

According to the severity of AD, we were unable to establish any association with the analyzed SNPs. The paucity of studies investigating this association underlines the need for further research in this area.

The prevalence of overweight, obesity, and dyslipidemia is higher in children with AD [23]. Moreover, 25 OH vitamin D deficiency may contribute to the development of hypertension, diabetes, hypertriglyceridemia, and obesity [30-32]. For this reason, we investigated the influence of 25 OH vitamin D deficiency and of SNPs in genes implicated in vitamin D metabolism (*VDR* and *CYP24A1*) on lipid parameters. rs2239185-AA, rs2239182-CC, and rs1540339-CC were associated with higher serum concentrations of total cholesterol, rs2239182-TT with a higher LDL cholesterol levels, and rs2239182-TC with lower HDL cholesterol levels. In all cases, cholesterol levels in the AD group were within the normal range; the observed difference were therefore quantitative, but unlikely to affect the individual’s health.

**Figure 1:**
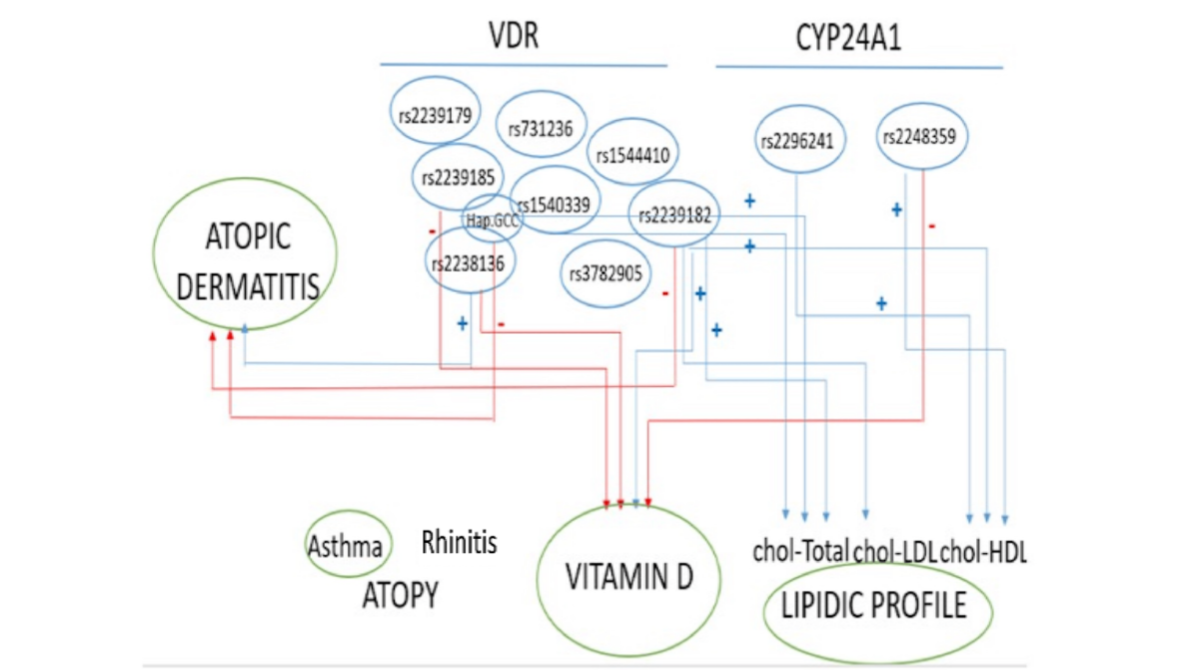
Summary of the main findings and associations between VDR and CYP24A1 polymorphisms and the variables analyzed. VDR: vitamin D receptor.

This study shows how the complicated genetic environment and interrelationships between different genetic factors influence the development of AD and of alterations in vitamin D metabolism or lipid profiles (Figure 1).

A global survey in 18 countries [33] revealed that AD affects around 20% of children, a substantial proportion of the pediatric population, although prevalence and severity varied across age groups and countries. Among the countries in Europe, Germany had the lowest prevalence (8.4%) and the Southern European countries of Spain and Italy had the highest prevalence (18.6% and 17.6%, respectively). A study led by Guttman-Yassky and Krueger [34] established, for the 6-7 years age group, an overall prevalence of 7.9%, and 6.2% in Spain. For the 13-14 years age group, the estimated prevalence was of around 15%. When stratified by sex, AD prevalence varied between male and female individuals, but no clear trend was observed. The estimation of prevalence by residential setting revealed that those living in rural areas had a lower prevalence of AD relative to urban or suburban settings.

It is well known that climate conditions have an influence in AD. Low temperatures and increased time spent indoors in warmth and low humidity increase the prevalence of AD. Increased ultraviolet radiation is inversely related to the prevalence of AD, as it exerts a protective effect because of its anti-inflammatory properties and increased production of vitamin D. The relief of Spain is characterized by being quite high, with an average altitude of 660 m above sea level. The varied orography of Spain, as well as its geographical location, in the middle latitudes of the temperate zone of the northern hemisphere means that the country has a remarkable climatic diversity. Thus, we pass from places with mild temperatures, around 15 °C, to others that exceed 40 °C in summer, and from places with a humid oceanic climate with annual rainfall of more than 2500 mm to places with a Mediterranean desert climate that do not exceed 200 mm per year. The thermal amplitude is greater in the interior of the Meseta, where it sometimes reaches 20 °C, whereas in places like the Canary Islands, this amplitude is smaller, and between the warmest and the coldest month, there is barely a variation of 5 °C.

There are other social determinants that affect in some way AD. The prevalence of AD has been found to increase with increasing socioeconomic status. The higher the socioeconomic status of parents, the higher the allergic sensitization and atopy in their children, probably related to the lower reduction in allergen exposure due to living in a better sanitary environment. Moreover, exposure to factors such as air pollution, tobacco, stress, or alcohol has been associated with a higher prevalence of AD.

25 OH vitamin D deficiency is a major health problem worldwide, especially in industrialized countries. As it is a problem that affects the general population, being not limited to atopic patients, public authorities have begun to promote safe sun exposure habits to maintain adequate 25 OH vitamin D serum concentrations. Our study evaluates, for the first time, the relationship between the *VDR* and *CYP24A1* polymorphisms and parameters related to phosphocalcic and vitamin D metabolism. Our findings shed light on the importance of genetic background, and not just exogenous VD intake, on the physiopathology of diseases in which this vitamin is implicated.

Some limitations of our study should be noted. First is the sample size. In addition, the patient and control cohorts were not exactly matched for age, weight, or height. This discrepancy could influence biochemical parameters. However, the reference values used in this study were equivalent across the age ranges of the population studied. Second, participants were recruited in the Departments of Dermatology and Paediatric Allergy of the San Jorge Hospital (Huesca, Spain), primary care centers in Huesca city, and the Dermatology Department of the Niño Jesús Hospital (Madrid, Spain). For this reason, the sample is not very representative of the whole country; ideally, more areas of Spain would have been covered during patient recruitment. According to the selection of patients, it must be noted that the high prevalence of AD complicates the process, as patients classified as healthy controls could feasibly become atopic over time. In addition, the generalizability of the findings is in some way compromised, because the study population included only Spanish individuals, and the genetic associations could be influenced by ethnicity. Finally, AD is a very heterogeneous disease in which the interplay between genomic changes associated with mutations in the key barrier and immune genes and a spectrum of environmental factors play a fundamental role in the pathogenesis. To make it more complex, recent studies indicate the importance of epigenetic alterations in the development of the disease. Epigenetic modifications are mainly mediated by DNA methylation, histone acetylation, and the action of specific micro-RNAs. It has been determined that the epigenome in patients with AD differs from the one observed in healthy individuals. This applies especially to the genes regulating immune responses and inflammatory processes, genes of the innate immunity, and those encoding the structural proteins of the epidermis [35].

In conclusion, these findings show how a complex genetic backdrop and interrelationships between different genetic factors influence alterations in vitamin D metabolism and lipid profiles and may contribute to the development of AD. We identified the SNP rs2238136 in *VDR* as a risk factor for AD and show that other SNPs in *VDR* and *CYP24A1* may lead to alterations in biochemical parameters and influence the risk of AD.

## Appendix

Multimedia Appendix 1 Genotype distributions of VDR and CY24A1 single nucleotide polymorphisms in the control and atopic dermatitis groups. Comparison with frequencies in populations in the 1000 Genomes Project.

## Abbreviations

AD: atopic dermatitis
HDL: high-density lipoprotein
LDL: low-density lipoprotein
OR: odds ratio
SCORAD: Scoring Atopic Dermatitis
SNP: single nucleotide polymorphism
VDR: vitamin D receptor gene
