# Supplementary material for: Association Between VDR and CYP24A1 Polymorphisms, Atopic Dermatitis, and Biochemical Lipid and Vitamin D Profiles in Spanish Population: Case-Control Study
Source: JMIR Dermatol. 2023 Jun 27;6:e39567. doi: 10.2196/39567 (PMC10337012; doi:10.2196/39567)
Supplement: Multimedia Appendix 1 [file derma_v6i1e39567_app1.docx]

Multimedia Appendix 1

Genotype distributions of VDR and CY24A1 single nucleotide polymorphisms in the control and atopic dermatitis groups. Comparison with frequencies in populations in the 1000 Genomes Project.

| **SNP** | **Gene** | **Ref Al** | **Alt Al** | **Genotype** | **Control** | | | | | **AD** | | | | ***1000 Genomes Project*** | | |
| --- | --- | --- | --- | --- | --- | --- | --- | --- | --- | --- | --- | --- | --- | --- | --- | --- |
|  |  |  |  |  | **n** | **F** | **MAF** | **HW** | **n** | | **F** | **MAF** | **HW** | **TOT** | **EUR** | **IBS** |
| **rs2239185** | **VDR** | G=0.498 | A=0.502 | A/A | 29 | 0.322 | 0.508 | 0.148 | 36 | | 0.319 | 0.527 | 0.428 | 0.277 | 0.340 | 0.374 |
|  |  |  |  | G/A | 30 | 0.337 |  |  | 47 | | 0.416 |  |  | 0.450 | 0.429 | 0.383 |
|  |  |  |  | G/G | 30 | 0.337 |  |  | 30 | | 0.265 |  |  | 0.273 | 0.231 | 0.243 |
| **rs1544410** | **VDR** | C=0.704 | T=0.296 | C/C | 27 | 0.380 | 0.585 | 0.404 | 40 | | 0.342 | 0.573 | 0.676 | 0.520 | 0.370 | 0.308 |
|  |  |  |  | C/T | 29 | 0.408 |  |  | 54 | | 0.462 |  |  | 0.367 | 0.433 | 0.505 |
|  |  |  |  | T/T | 15 | 0.211 |  |  | 23 | | 0.197 |  |  | 0.112 | 0.177 | 0.187 |
| **rs2238136** | **VDR** | C=0.833 | T=0.167 | C/C | 50 | 0.676 | 0.639 | 0.148 | 45 | | 0.433 | 0.605 | 0.625 | 0.700 | 0.527 | 0.505 |
|  |  |  |  | C/T | 18 | 0.243 |  |  | 51 | | 0.490 |  |  | 0.267 | 0.408 | 0.449 |
|  |  |  |  | T/T | 6 | 0.081 |  |  | 8 | | 0.077 |  |  | 0.034 | 0.066 | 0.047 |
| **rs3782905** | **VDR** | G=0.763 | C=0.237 | G/G | 47 | 0.540 | 0.713 | 0.404 | 66 | | 0.555 | 0.739 | 0.691 | 0.589 | 0.455 | 0.421 |
|  |  |  |  | G/C | 30 | 0.345 |  |  | 44 | | 0.370 |  |  | 0.347 | 0.421 | 0.467 |
|  |  |  |  | C/C | 10 | 0.115 |  |  | 9 | | 0.076 |  |  | 0.064 | 0.123 | 0.112 |
| **rs2239179** | **VDR** | T=0.639 | C=0.361 | T/T | 36 | 0.448 | 0.647 | 0.532 | 48 | | 0.425 | 0.664 | 0.676 | 0.428 | 0.354 | 0.290 |
|  |  |  |  | T/C | 32 | 0.397 |  |  | 54 | | 0.478 |  |  | 0.422 | 0.431 | 0.495 |
|  |  |  |  | C/C | 12 | 0.155 |  |  | 11 | | 0.097 |  |  | 0.150 | 0.211 | 0.215 |
| **rs1540339** | **VDR** | C=0.614 | T=0.386 | C/C | 32 | 0.397 | 0.610 | 0.544 | 44 | | 0.379 | 0.582 | 0.428 | 0.419 | 0.412 | 0.449 |
|  |  |  |  | T/C | 35 | 0.426 |  |  | 47 | | 0.405 |  |  | 0.390 | 0.439 | 0.439 |
|  |  |  |  | T/T | 15 | 0.183 |  |  | 25 | | 0.216 |  |  | 0.191 | 0.149 | 0.112 |
| **rs2239182** | **VDR** | T=0.521 | C=0.479 | T/T | 24 | 0.282 | 0.506 | 0.532 | 45 | | 0.375 | 0.592 | 0.554 | 0.292 | 0.252 | 0.159 |
|  |  |  |  | T/C | 38 | 0.447 |  |  | 52 | | 0.433 |  |  | 0.459 | 0.475 | 0.561 |
|  |  |  |  | C/C | 23 | 0.271 |  |  | 23 | | 0.192 |  |  | 0.250 | 0.272 | 0.280 |
| **rs731236** | **VDR** | A=0.723 | G=0.277 | A/A | 37 | 0.475 | 0.639 | 0.148 | 44 | | 0.386 | 0.605 | 0.625 | 0.544 | 0.378 | 0.327 |
|  |  |  |  | G/A | 25 | 0.328 |  |  | 50 | | 0.439 |  |  | 0.360 | 0.445 | 0.486 |
|  |  |  |  | G/G | 15 | 0.197 |  |  | 20 | | 0.175 |  |  | 0.097 | 0.177 | 0.187 |
| **rs2248359** | **CYP24A1** | C=0.551 | T=0.449 | C/C | 33 | 0.344 | 0.531 | 0.404 | 35 | | 0.360 | 0.564 | 0.554 | 0.313 | 0.344 | 0.374 |
|  |  |  |  | C/T | 36 | 0.375 |  |  | 40 | | 0.412 |  |  | 0.476 | 0.499 | 0.495 |
|  |  |  |  | T/T | 27 | 0.281 |  |  | 22 | | 0.226 |  |  | 0.211 | 0.157 | 0.131 |
| **rs2296241** | **CYP24A1** | G=0.542 | A=0.458 | G/G | 17 | 0.170 | 0.603 | 0.811 | 22 | | 0.244 | 0.524 | 0.754 | 0.292 | 0.211 | 0.252 |
|  |  |  |  | G/A | 44 | 0.440 |  |  | 43 | | 0.463 |  |  | 0.499 | 0.513 | 0.449 |
|  |  |  |  | A/A | 39 | 0.390 |  |  | 27 | | 0.293 |  |  | 0.208 | 0.276 | 0.299 |

Abbreviations: AD, atopic dermatitis; Ref Al, reference allele; Alt Al, alternative allele; F, frequency; MAF, minor allele frequency; HW, p-value for Hardy-Weinberg equilibrium test; SNP, single nucleotide polymorphism. Sample size (n) varies according to the number of valid samples in each molecular assay. Frequency of 1000 Genomes Project Phase 3 populations: TOT (global, n=2504); EUR (European, n=503); IBS (Iberian, n=107).
